# Identification of Lipopeptide Iturin A Produced by *Bacillus amyloliquefaciens* NCPSJ7 and Its Antifungal Activities against *Fusarium oxysporum* f. sp. *niveum*

**DOI:** 10.3390/foods11192996

**Published:** 2022-09-26

**Authors:** Junhua Wang, Jiying Qiu, Xiaoyu Yang, Jinyu Yang, Shuangzhi Zhao, Qingxin Zhou, Leilei Chen

**Affiliations:** Key Laboratory of Agro-Products Processing Technology of Shandong Province, Key Laboratory of Novel Food Resources Processing, Ministry of Agriculture and Rural Affairs, Institute of Agro-Food Science and Technology, Shandong Academy of Agricultural Sciences, Jinan 250100, China

**Keywords:** *Bacillus amyloliquefaciens*, iturin A, ultrastructure, ergosterol, fungicidal activities, *Fusarium oxysporum* f. sp. *niveum*

## Abstract

*Bacillus amyloliquefaciens* NCPSJ7 showed potential fungicidal activities for the effective control of fungal infection. From the PCR test, the key genes (*srfAA*, *sfp*, *fenD*, *bmyB*, *ituD*, and *ituC*) were detected in *B. amyloliquefaciens* NCPSJ7. These genes were closely related to the lipopeptides (LPs) synthesis. Next, three LPs families were identified with liquid chromatography–mass spectrometry (LC/MS), including iturin A, fengycin A, and surfactin. After purification with C18, the main active antifungal compound was proven to be C14-iturin A by ESI-HRMS, which has significant activities against fungi. These results proved that C14-iturin A played an important role in inhibiting the growth of fungi for *B. amyloliquefaciens* NCPSJ7. Furthermore, the isolated LP could inhibit mycelial growth and conidia germination at 30 μg/mL. SEM allowed us to observe that mycelial morphology and conidia germination were also affected. The mycelial ultrastructure TEM observations showed that the external electron-dense outer layer cell wall, which mainly consisted of glycoproteins, was affected. Furthermore, swollen mitochondria, enriched glycogen, and increased vacuoles were also found. LP also affected the intact wall and membranes, leading to their increased permeability, which was proved by propidium iodide (PI) staining and conductivity measurements. Meanwhile, the ergosterol, which has an affinity for iturin A, also increased. These results indicated that LP caused fungal dysfunction and membrane permeability increase, leading to fungal inhibition. Identifying and studying LPs is important in exploring the fungicidal activities of *B. amyloliquefaciens*, which promotes the use of *B. amyloliquefaciens* NCPSJ7 as a potential candidate for biocontrol.

## 1. Introduction

*Bacillus amyloliquefaciens* is a member of the free-living soil bacteria known to promote plant growth and suppress plant pathogens; it is also used as a commercial biopesticide and biofertilizer [1]. When the whole-genome sequencing of *B. amyloliquefaciens* FZB42 was reported in 2007, it paved the way for more antimicrobial-related gene clusters to be found in *B. amyloliquefaciens*, attracting the attention of more scientists studying the strain as a form of biocontrol [2,3]. Among the secreted antimicrobial substances, lipopeptides (LPs), such as bacillomycin D, surfactin, iturin, and fengycin, have been mostly studied for their antagonistic activities [4]. They have a well-recognized potential to be used in biocontrol because of their antifungal activities and surfactant properties. LPs could also induce the defense responses to conquer pathogens [5,6], thus supporting the active substances to be developed as biocontrol agents [7,8]. Otherwise, LPs have been shown to control resistant bacteria and have a potential function in the prevention of bacterial resistance [9].

For some potent antagonistic bacteria, the presence of *fenD* has been associated with the production of various species of fengycin isoforms, while the presence of *ituC* has been related to an iturin subtype. Remarkably, the presence of *srfAA* has facilitated strong antibacterial activities, along with the growth media. The relationship mainly exists in the species *B. subtilis* and *B. amyloliquefaciens* [10,11]. Furthermore, 4-phosphopantetheinyl transferase *sfp* gene has been found to play a critical role in iturin A and surfactin secretion in *B. subtilis* RB14 [12,13]; thus, it would be convenient to identify and separate the active compounds with the understanding of the gene markers [14]. The combined study with polymerase chain reaction (PCR) amplification and mass spectrometer technology is effective for investigating LPs [15,16].

Our previous study identified a potent antagonistic *B. amyloliquefaciens* NCPSJ7 with strong inhibition of the growth of various pathogenic fungi [17]. Furthermore, the strain could protect postharvest grapes from infection caused by *Botrytis cinerea* [18]. After fermentation, an active peptide AFP3 was separated from the supernatant, which showed potent activities against a broad spectrum of fungi with strong stability, especially in *Fusarium oxysporum* [17]. *F. oxysporum* is a deadly pathogen that infects agricultural products. Once established in the soil, it is difficult to eradicate because the thick spores remain dormant and infect the soil for many years [19]. *F. oxysporum* f. sp. *niveum* (FON) is an important pathogen, which permanently threatens the growth of watermelon. Chemical fungicides are used to control the fungus, which are not friendly to the environment and can cause fungicide resistance in FON [20,21]. In order to achieve potential fungicidal activities, low environmental hazards, and low propensity to induce tolerance, antimicrobial peptides were studied to reduce or replace the chemical fungicides. Moreover, lipopeptides guided chemosensitization of *Fusarium* sp. to chemical fungicides [22].

Then, the active peptide AFP3 was identified by MALDI-TOF-MS. Molecular mass ions [M+H]^+^ at *m/z* 1463.8018, 1477.8185, 1491.8347, 1505.8503, and [M+2H]^2+^ at *m/z* 732.4041, 739.4125, 746.4200, 753.4282 were found (Figure S1), which were assigned to fengycin, a potential fungicidal LP [23]. These results showed the NCPSJ7 strain secreted LPs, which could be responsible for inhibiting the growth of fungi. Therefore, the other potential LPs were explored in the study. This study aimed to identify the decisive fungicidal ingredients and their mechanisms of action against *Fusarium oxysporum* f. sp. *niveum* (FON), which provide a good foundation for the practical applications of the LPs and *B. amyloliquefaciens* NCPSJ7 in the future.

## 2. Materials and Methods

### 2.1. Microorganisms and Culture Conditions

The strain *B. amyloliquefaciens* NCPSJ7 was isolated from a ginger field and then deposited in the China Center for Type Culture Collection (CCTCC No. M 2013098). *Bacillus* sp. 222 was screened and preserved in the laboratory. The cultured medium contained 0.5% glucose, 0.75% yeast extract, 0.75% tryptone, 0.5% (NH_4_)_2_SO_4_, and 0.5% NaCl (pH 7.0). Pathogenic fungi FON were purchased from the Agricultural Culture Collection of China (ACCC No. 30024) and incubated in a potato dextrose agar medium (PDA, pH 5.6) containing 0.6% potato extract, 2% glucose, and 2% agar (Qingdao Hope Bio-Technology Co., Ltd., Qingdao, China). No agar was present in the PDB broth. The standard for iturin A was purchased from Sigma-Aldrich (Burlington, MA, USA). LPs were purified by C18 silica gel chromatography (40–60 µm). The electron microscopy fixative was purchased from the Beijing Solarbio Science and Technology Co., Ltd. (Beijing, China).

### 2.2. LPs Gene PCR

The genomic DNA of *B. amyloliquefaciens* NCPSJ7 and *Bacillus* sp. 222 was isolated and purified with a Wizard^®^ genomic DNA purification kit according to the manufacturer’s instructions. The genomic DNA of *B. subtilis* 168 and *Clostridium* sp., which was used as negative control, was kindly provided by Prof. Yi Wang’s lab from Auburn University. PCR was carried out in a total volume of 25 µL containing 12.5 µL of GoTaq^®^ Green Master Mix (2×), 0.5 μM of each primer (Table 1), 1 µL of genomic DNA, and ddH_2_O. The PCR conditions were as follows: 95 °C for 5 min, 30 cycles of 95 °C for 30 s, annealing temperature for 1 min, 72 °C for 1 min, and then, an extension step at 72 °C for 5 min followed by a 4 °C soak.

The PCR products were analyzed in 1% agarose gel (with 50 µL ethidium bromide per 1 L) in 1× Tris-acetate EDTA (TAE) with a Quick-Load^®^ 100 bp DNA ladder (Biolabs^®^), running for 18 min at 180 V. Gel images were captured on an Alpha Innotech AlphaImager™ Gel Imaging System.

### 2.3. LPs Crude Extraction and Purification

As described previously [17], *B. amyloliquefaciens* NCPSJ7 was incubated at 33 °C at a centrifugation rate of 150 rpm for 6 days. After centrifugation, the supernatant was acidified with 6M HCl to pH 2.0 [26]. After 4 h, the precipitate was extracted with methanol thrice. After rotary evaporation, the residue was dissolved in 50 mM Tris-HCl buffer (pH 7.5) to obtain LPs crude.

Subsequently, LPs crude extract was purified with C18 chromatography (Boshi Bio-tech, Shanghai, China, 15 mm × 250 mm) using a gradient elution of 20% (*v*/*v*), 40%, 60%, 80%, and 100% methanol. The antifungal activities to FON were tested. The active elution was collected and analyzed.

### 2.4. High-Performance Liquid Chromatography (HPLC) Analysis and Liquid Chromatography–Mass Spectrometry (LC/MS) Analysis

LPs were detected on an Agilent 1260 HPLC instrument using an Agilent ZORBAX SB-C18 column (5 µm, 4.6 mm × 150 mm), eluted with methanol and water as the mobile phase, with DAD detection at 220 nm and a flow rate of 1.0 mL/min. The sample injection volume was 10 μL. The column was equilibrated with 20% (*v*/*v*) methanol. The gradient conditions were as follows: (ddH_2_O (A) and methanol (B)) 0–2 min, 40% B; 2–12 min, 60% B; 12–22 min, 80% B; 22–25 min, 100% B; and 25–30 min, 100% B. The post-run time was set at 5 min.

LC/MS analysis was performed on a rapid resolution liquid chromatography–quadrupole-time-of-flight mass spectrometer (1200RRLC-6520 Accurate Mass Q-TOF LC/MS, Agilent) with electrospray ionization (ESI) in positive mode. High-resolution mass spectral (HRMS) data were displayed as *m/z* on an Agilent 6520 Quadrupole Time-of-Flight LC/MS delivered in positive and negative modes.

### 2.5. Antifungal Assays

With modified methods based on the previous protocols [17], the antifungal activities of the elution were tested. A piece of activated pathogen (FON) was resuspended in sterile water. The conidial suspension (50 µL/plate) was spread onto a fresh PDA plate (6 cm, containing 30 μg/mL chloramphenicol in it). Subsequently, 100 µL analyte was placed onto the pathogen plate. Then, the plates were cultured at 28 °C for 48 h.

### 2.6. Determination of the Dry Weight of FON

The inhibition test was conducted according to the procedure detailed in a previous work [27], with minor modifications. FON was grown in PDB at 28 °C, 155 rpm. After 7 days, the conidia were collected and adjusted to 10^8^/mL. An amount of 1 mL conidia was added to 100 mL PDB (containing chloramphenicol with a final concentration of 30 µg/mL). The PDB also contained LP (30, 60, 90, 120, and 150 µg/mL, LP treated group, T). The group with phosphate-buffered saline (PBS) instead of LP was set as blank control (C_0_). The mixture was cultured for 7 days on a shaker at 155 rpm at 28 °C. Then, the mycelium was collected by filtration and washed twice with distilled water. Afterward, the pellets were dried in a vacuum drying incubator at 40 °C overnight and weighed. The median effective concentration (EC_50_) of LP was calculated based on the linear regression of the inhibition ratio (I) of the log-transformed fungicide concentration given by

Inhibition ratio of dry weight = (C_0_ − T)/C_0_ × 100% (1)

### 2.7. Scanning Electron Microscopy (SEM) and Transmission Electron Microscopy (TEM)

The harvested conidia grew at 28 °C, 155 rpm, for 24 h. Then, the LP was added to a final concentration of 60 µg/mL for 6 or 24 h. Next, the mycelia were collected and washed with water and then fixed by the electron microscopy fixative (2.5%, Solarbio, Beijing, China) for 4 h at room temperature. The sample using PBS instead of LP was used as control. Subsequently, the samples were transferred into 4 °C for preservation and transportation. For conidia, they grew at 28 °C, 155 rpm, for 6 h with LP concentrations of 30, 60, 90, 120, and 150 µg/mL. The samples were treated the same as the above.

The staining and analysis were conducted by Wuhan Servicebio Technology Co., Ltd. (Wuhan, China). The process was as follows. After being fixed with 1% OsO_4_ in 0.1 mol/L PBS (pH 7.4) for 2 h at room temperature, the samples were successively dehydrated with increasing concentrations of ethanol and isoamyl acetate. The dehydrated samples were dried to the critical point, coated with gold, and observed using an SU8100 SEM (Hitachi Ltd., Tokyo, Japan).

For TEM, pre-embedding with 1% agarose was used before being fixed with 1% OsO_4_ and dehydrated. Then, resin penetration was conducted using acetone and EMBed 812 from 1:1 to 1:2 and embedded with pure EMBed 812. After polymerization at 65 °C, the sample was cut to 60–80 nm and fished out onto the 150 meshes cuprum grids with Formvar film. After that, the samples were stained with 2% uranyl acetate and 2.6% lead citrate. The grids were observed under a HT7700 transmission electron microscope (Hitachi Ltd., Tokyo, Japan).

### 2.8. Membrane Conductivity [21]

The mycelia were collected by filtration after the conidia were shaken at 180 rpm at a temperature of 28 °C for 48 h. Then, 0.3 g of mycelia were suspended in 40 mL of distilled water, which contained LP at the EC_50_ (60 µg/mL). The group without LPs was used as control. The conductivity of the treated water was measured after 6, 12, 18, 24, 30, 36, 42, and 48 h with a conductivity meter (OHAUS^®^ STARTER3100C, Parsippany, NJ, USA).

### 2.9. Measurement of Membrane Permeability

Membrane permeability was determined by the uptake of propidium iodide (PI) according to the reported method, with minor modification [28]. The harvested conidia grew in PDB at 28 °C at 155 rpm for 24 h. Then, the LP was added to a final concentration of 60 µg/mL for another 6 h. Subsequently, the cells were washed with and resuspended in 1 mol/L PBS (pH 7.2) and then incubated with 50 μg/mL PI in the dark for 20 min at room temperature. The fluorescence images were obtained with a fluorescence microscope (Olympus IX71S1F-3). The mycelia without LP treatment were used as control.

### 2.10. Ergosterol Content

The mycelium was incubated and treated, as mentioned in Section 2.7, with 60 µg/mL LP for 6 h and 24 h, respectively. Then, the mycelium was collected, freeze dried, weighted, and ground to a fine powder. Intracellular ergosterol content was analyzed according to a previously reported method [29]. The mycelium powder underwent saponification with 10% NaOH mixed with methanol in a water bath at 90 °C. One hour later, 95% ethanol was added for another hour. Then, 2 mL ddH_2_O and 5 mL n-heptane were added. After vigorous vortex agitation and centrifugation at 4000 rpm for 15 min, the supernatant was diluted with 95% ethanol to obtain absorbance at 282.5 nm using a hybrid plate reader (Synergy H1M, BioTek, Winooski, VT, USA). The ergosterol content was calculated according to the standard curve, which was built by measuring the absorbance of ergosterol solutions at concentrations from 0.625 to 80 mg/L.

### 2.11. Statistical Analysis

All experiments were conducted independently at least twice. Data were analyzed with GraphPad Prism version 6.0. Differences between the groups were analyzed using the Duncan test in the SPSS Statistic 22 software. A *p*-value below 0.05 or 0.01 was considered a statistically significant difference.

## 3. Results

### 3.1. PCR of LP Biosynthetic Genes

**Figure 1:**
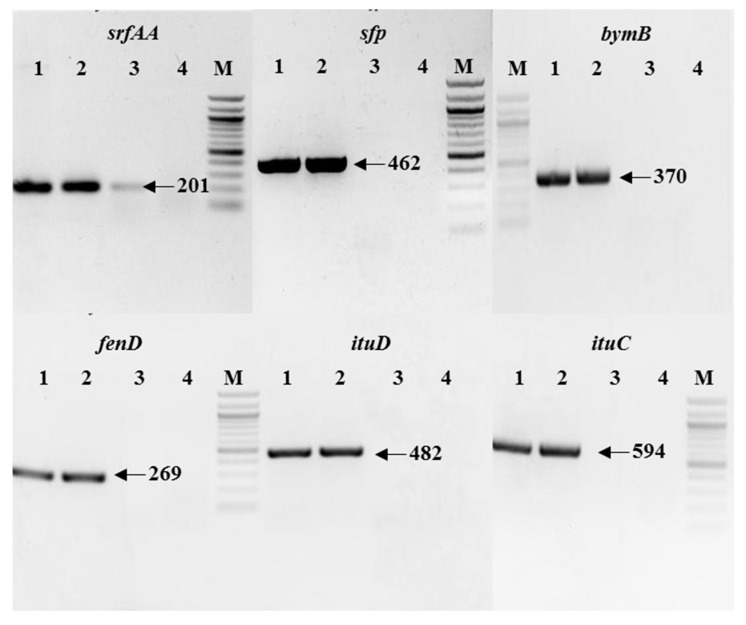
Agarose gel electrophoresis of PCR products for surfactin (*srfAA*), fengycin (*fenD*), bacillomycin (*bymB*), iturin (*ituC* and *ituD*), and *sfp* (essential for various LPs synthesis). Lanes 1–4: The biosynthesis genes from *B. amyloliquefaciens* NCPSJ7, *Bacillus* sp. 222 (The whole genome was analyzed and acted as positive control. Data were not shown here.), *B. subtilis* 168, and *Clostridium* sp. (Negative control). M: 100 bp DNA ladder (Biolabs^®^). The product size as designed was indicated with arrow.

The genes related to LPs biosynthesis (*srfAA*, *sfp*, *bmyB*, *fenD*, *ituC*, and *ituD*) were tested in the PCR analysis. From the PCR results, *B. amyloliquefaciens* NCPSJ7 was shown to hold these genes (Figure 1), indicating that NCPSJ7 might show antagonistic activities by secreting LPs (surfactin, fengycin, bacillomycin, or iturin).

### 3.2. The Purification and Characterazation of the Fungicidal LPs

Using the LC/MS method, three types of LPs were examined from LPs crude extract, including surfactin, iturin A, and fengycin A (Table 2, Figure S2). After purification with C18 chromatography, the elution of 80% methanol showed inhibitory activities to the growth of the FON. From the HRMS results, the *m/z* values of major ions were 1043.5374, 1057.4215, and 1079.5530, which were assigned to C14-iturin A [M + H]^+^, C15-iturin A [M+H]^+^, and C15-iturin A [M + Na]^+^, respectively [30]. The active component was thought to consist of iturin A homologs. The iturin A standard was purchased and analyzed with HPLC. The peaks of the elution of 80% methanol were identical with those of iturin A standard (Figure S3), demonstrating that iturin A played an important role in the fungicidal activities of the strain NCPSJ7.

For confirmation, a second chromatography with C18 was performed. HRMS showed ion peaks 1043.5247 [M + H]^+^ and 1065.5057 [M + Na]^+^ in the positive mode, as well as 1041.4781 [M − H]^−^ and 1077.4528 [M + Cl]^−^ in the negative mode (Figure S4). The results revealed iturin A as the main anti-fungi component, which dominated the fungicidal activities and should be tested for inhibitory activities against FON in subsequent studies.

### 3.3. Effect of Iturin A Treatment on the Growth of FON Mycelia

**Figure 2:**
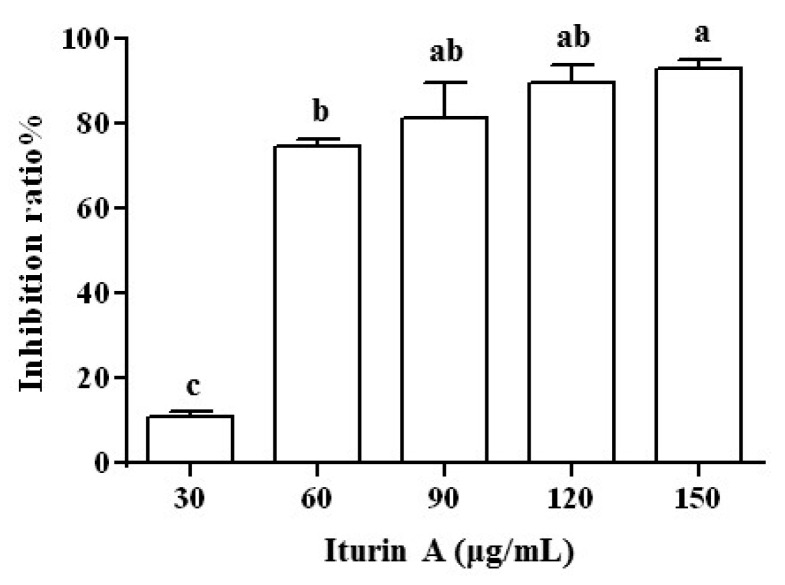
Effect of iturin A at different concentrations on the dry weight of FON mycelia. Different lowercase letters indicate significant differences (*p* < 0.05).

The inhibition ratios of the dry weight of the FON mycelia were measured at various concentrations of iturin A, as shown in Figure 2. As can be seen, as the concentration of iturin A increased from 30 to 150 μg/mL, the inhibition ratio increased with it. An EC_50_ of 60 µg/mL was obtained, which was defined as the concentration of the drug added to the culture that reduced the density of the mycelia to 50% of the control group in vitro.

### 3.4. SEM and TEM Observation of Mycelia and Conidia

**Figure 3:**
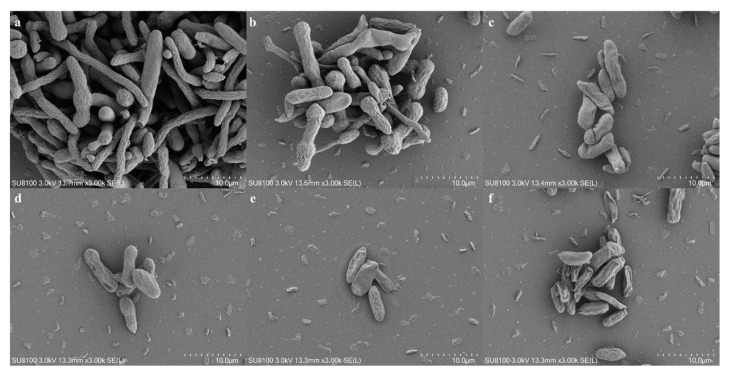
Morphological change of FON conidia exposed to various concentrations of iturin A. (**a**) 0 μg/mL, control; (**b**) 30 μg/mL; (**c**) 60 μg/mL; (**d**) 90 μg/mL; (**e**) 120 μg/mL; (**f**) 150 μg/mL.

Before SEM observation, the conidia were treated with iturin A at a range of concentrations for 6 h. The results showed that the conidia germinated mostly with replete and smooth surfaces in the control group. After treatment, the conidial germination was partly inhibited by iturin A at 30 μg/mL, with slight surface indentation (Figure 3). For the other treated groups, the shrinkage deformation became worse as the concentration of iturin A increased, which possibly referred to the death of cells caused by iturin A.

**Figure 4:**
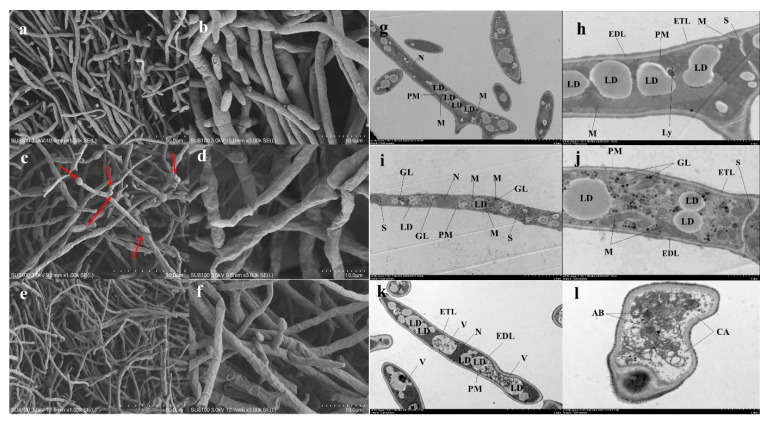
Ultrastructural changes in mycelia being treated with iturin A (60 μg/mL). (**a**,**b**,**g**,**h**) control group; (**c**,**d**,**i**,**j**) mycelium treated with iturin A for 6 h; (**e**,**f**,**k**,**l**) mycelium treated with iturin A for 24 h. V, vacuole; PM, plasma membrane; S, septum; EDL, external electron-dense outer layer cell wall; ETL, underlying electron-transparent layer; LD, lipid droplets; GL, glycogen; Ly, lysosome; M, mitochondria; CA, a large cavitation area; N, Nucleus; AB, autophagic bodies.

The SEM results showed that the mycelium was dense and filled with smooth surfaces in the control group. After being treated for 6 h, the mycelium deformed slightly. Mycelial swelling occurred locally (red arrow, Figure 4c). The mycelium was rough and uneven on the surface (Figure 4d). The mycelium morphology changed more severely at 24 h and became significantly thinner. The shrinkage deformation also worsened (Figure 4e,f). The damage also occurred in the cell ultrastructures according to the TEM images. In the control group, the normal mycelial cell wall, including the external electron-dense outer layer cell wall (EDL) and an underlying electron-transparent layer (ETL), along with the plasma membranes, were intact with equal widths [31]. The cytoplasm showed an even distribution of abundant organelles. The organelles were distributed in a normal arrangement, along with the septum, with uniform composition and structure that spanned the entire width of the hyphae. After being treated for 6 h, the EDL became thinner, proving that iturin A could damage the FON cell wall.

The role of cell wall degradation in inhibiting the growth of *Fusarium* spp. has been reported previously [32]. The well-distributed cytoplasmic content became sparse and light at 6 h. The mitochondria was enlarged mostly with clear ridges. At 24 h, larger vacuoles appeared. Most organelles were even squeezed to the cytoplasmic edge. The TEM images also confirmed the formation of autophagic bodies in some cells (Figure 4l). Some organelles were invisible, including lipid droplets, mitochondria, nucleus, and so on. Finally, the ETL was still intact even after 24 h.

### 3.5. Effects of Iturin A on Plasma Membrane Permeability

**Figure 5:**
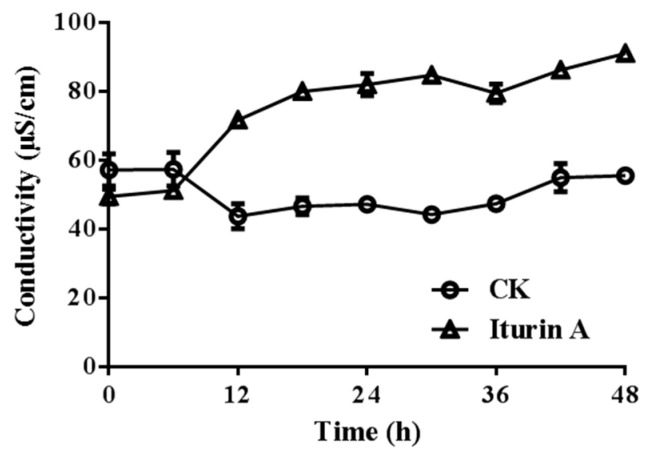
Effect of iturin A on extracellular conductivity of mycelia at the concentration of EC_50_.

Electrical conductance reflects the permeability and integrity of a membrane [21]. The extracellular mycelial conductivity was monitored to confirm the membrane disruption effects of iturin A. As shown in Figure 5, the conductivity increased after 6 h incubation. Then, 6 h later, the conductivity increased sharply, slowed down, and then remained relatively stable until 48 h. The results showed that iturin A affected FON extracellular conductivity.

**Figure 6:**
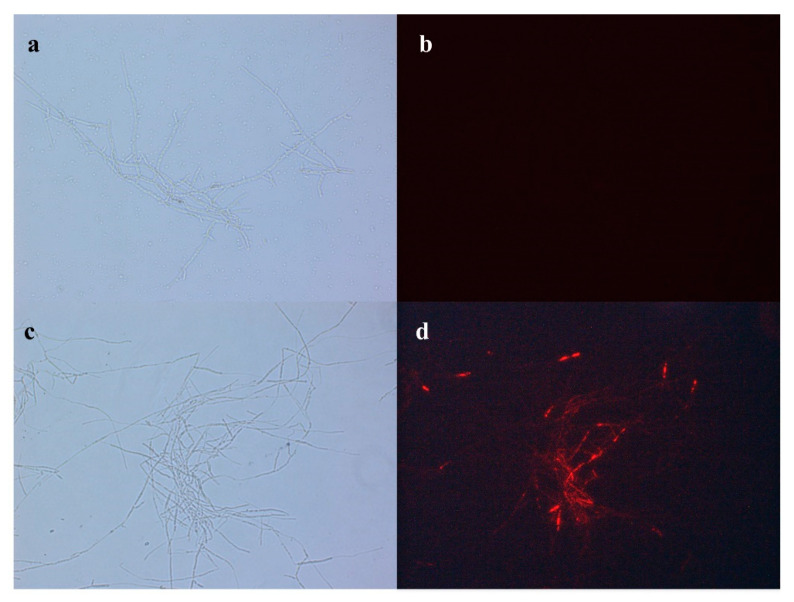
Effects of iturin A on membrane permeability of mycelia. Optical microscope, (**a**,**c**); Fluorescence microscope, (**b**,**d**); Control, **a**,**b**; Treatment, **c**,**d**, being treated with iturin A at a concentration of 60 μg/mL.

The PI uptake assay was used to detect the effects of iturin on the permeability of the membrane. PI cannot penetrate the membrane unless it is damaged. As shown in Figure 6b, the mycelia were not stained with fluorescence. Otherwise, several treated mycelia were stained with red fluorescence (Figure 6d), indicating that iturin A could increase the membrane permeability.

### 3.6. Effects of Iturin A on Ergosterol Content

**Figure 7:**
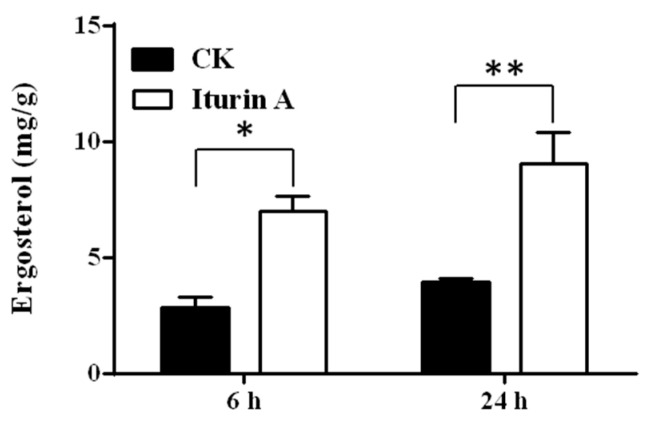
Effects of iturin A on the ergosterol content of mycelia at 6 h and 24 h. *, significantly different at *p* < 0.05; **, significantly different at *p* < 0.01.

Ergosterol is the essential component of cell membranes in fungi and plays a key role in membrane-bound enzyme activity, membrane fluidity, membrane integrity, and substrate transport, mediating the permeability of fungal membranes [29,33]. Then, the ergosterol content of mycelia was analyzed to find out the reasons leading to the increased permeability. Compared with untreated mycelia, the ergosterol content increased in the treated group at 6 h (2.80 vs. 6.99 mg/g) and 24 h (3.88 vs. 9.05 mg/g), as shown in Figure 7. Notably, the ergosterol content gradually increased over time.

## 4. Discussion

Based on the previous study, *B. amyloliquefaciens* NCPSJ7 may have reduced the spoilage of apples and pears by secreting active peptides to control the rotting [17]. For most *Bacillus* spp., LPs have been shown to be the key factors inhibiting fungi growth [34,35]. Thus, in the current study, LPs may have played important roles in NCPSJ7′s fungi control. Therefore, the purification, identification, and fungicidal mechanisms of LPs were investigated in the study.

The antagonistic capacity of *Bacillus* spp. against pathogens is related to the presence of LPs genes and to the production of the corresponding LPs, *srfAA* (surfactin), *fenD* (fengycin), *bmyB* (bacillomycin), and *ituC* and *ituD* (iturin), which is mainly associated with the species *B. subtilis* and *B. amyloliquefaciens* [10,11]. Sixty-four strains of *Bacillus* spp. isolates were studied, and the results showed that the most common antimicrobial peptide genes were *bmyB*, *srfAA*, and *fenD* (34–50% of isolates). Meanwhile, most isolates (98.4%) yielded surfactin isomers, 90.6% iturins, and 79.7% fengycins. Therefore, the most common LPs biosynthetic-related genes were investigated and detected in the study. Combined with PCR analysis, the LC/MS analysis proved that NCPSJ7 secreted LPs, including surfactin, iturin A, and fengycin A, responsible for fungal inhibition. After fractional purification, C14-iturin A was proven to be the dominant LP. As a member of the iturin family, iturin A has strong antifungal effects and a broad antifungal spectrum, showing the potential fungicidal activities to be applied as biocontrol.

Iturin A, which has long been found to inhibit pathogenic fungi, is mainly secreted by *Bacillus* spp. Recently, C15-iturin A (iturin A5) secreted from thermotolerant marine *B. amyloliquefaciens* has been reported to have a significant effect on *F. oxysporum* [36]. In this study, iturin A proved to inhibit conidia germination and caused mycelium damage to FON with the increase in LP concentration and the passage of time. The inhibitory activities on conidia were similar to the effect of iturin A5 on *Fusarium oxysporum f.* sp. *cubense*. At 125 µg/mL, iturin A5 caused germ tube distortion and depolarization, and iturin A caused the same effect at 60 µg/mL.

For mycelium, the conductivity of mycelium increased after iturin A treatment in the study. Other authors also reported that the conductivity changes were related with the increased permeability of the membrane [21]. Moreover, PI penetrated cells with damaged membranes and emitted red fluorescence. The mycelium was stained by PI, which showed that the membrane was destroyed after treatment with LP. Through TEM, the irregular organelle shape with unclear boundaries was observed in this study. Additionally, glycogen accumulated significantly in the cytosolic after being treated for 6 h. No large vacuole was found until 24 h, and the rest of the vacuoles showed glycogen concentrations inside them. According to a previous report, a differential transcriptome analysis showed that iturin A inhibited *Aspergillus carbonarius* by changing the fungal cell structure and causing energy, transport, and osmotic pressure metabolism disorder [37]. Moreover, autophagic bodies were found by TEM analysis in the study, which revealed that autophagy might have occurred in the treated mycelium. According to the reports, glycogen accumulated when the yeast was exposed to nutrient-limited conditions [38]. The vacuoles played a key role in the long-term maintenance of glycogen and glycogen metabolism [38], which increased with the glycogen accumulation in this study. Similar to glycogen accumulation, autophagy occurs when there are nutrient deficiencies in yeast [38].

In the current study, mitochondrial swelling occurred in the FON mycelium being treated with iturin A. A report indicated that mitochondrial swelling might be related to abnormal energy metabolism, which affects multicellular organisms and triggers apoptosis [39]. In fact, apoptosis was observed in *Candida* *albicans* through membrane permeability, ROS accumulation, mitochondrial transmembrane potential, and chromatic agglutination [28]. However, in the current study, no apoptosis signature was observed in FON mycelium by Annexin V-FITC/PI, JC-1 and DAPI dihydrochloride staining (data not shown here). The results indicated that iturin A did not cause apoptosis in FON mycelium, but autophagy, which may be closely related to the abnormal function.

Ergosterol plays an important role in membrane fluidity and membrane integrity [20,40]. Thus, ergosterol content was analyzed in this study, and the results showed that the treated mycelia had more ergosterol. Iturins can interact with ergosterol in the cytoplasmic membrane, leading to the formation of ion-conducting pores and the outflow of intracellular ions, such as K^+^ [36,41]. The growing ergosterol might increase membrane affinity. However, the growing ergosterol contradicted with increased cell membrane permeability. No experiments indicated that iturin A can induce the increase in ergosterol before, apart from transcriptome analysis [42]. According to the report, the ergosterol synthesis key genes, *Erg28* and *Cyp51* (*Erg11*), were significantly upregulated in the *Sclerotinia sclerotiorum*, which was treated with *B. amyloliquefaciens*, producing cyclic LPs for fungal inhibition. It has been assumed that the fungi might upregulate ergosterol synthesis to respond to the stress of membrane permeability increase [42]. This hypothesis should be tested and verified in a further study.

Meanwhile, iturin A caused substantial structural destruction in the FON cell wall. The treated cell walls had no discernible layers and showed uneven widths with thin or gapped structures. The defects in cell wall integrity were also observed in *Verticillium dahliae* [43]. In this study, the morphological observations revealed that iturin A made the cell wall uneven not on ETL but on EDL, which is rich in glycoproteins. In yeast, cell wall glycoproteins determined the permeability of the wall [44] and prevented the infection of plants by pathogenic fungi [45]. Accordingly, iturin A might affect glycoprotein synthesis to inhibit the growth of FON and prevent fungal infection. Few LPs were also found to disrupt the EDL of FON. For ETL, it was not affected at all. The underlying ETL was enriched with carbohydrates, probably including β-1,3-glucan, and chitin, representing the skeletal layer [31,44].

## 5. Conclusions

Key genes (*srfAA*, *sfp*, *fenD*, *bmyB*, *ituD*, and *ituC*) related to LP synthesis were detected in the genome of *B. amyloliquefaciens* NCPSJ7. Using HPLC/MS, three families of LPs were identified in the fermentation supernatant, including iturin A, fengycin A, and surfactin. After purification with reverse C18 column, C14-iturin A was proven to be the main factor inducing the potent fungicidal activities of NCPSJ7. Morphological observations revealed that iturin A prevented the germination of fungal conidia. The integrity of the cell wall and membrane was destroyed. Moreover, cell dysfunction was supported by mitochondrial swelling, glycogen accumulation, and autophagic bodies. Furthermore, ergosterol on cell membranes increased. How iturin A interferes with the glycoproteins to affect cell wall integrity should be further investigated in future studies.

## Figures and Tables

**Table 1:** Primers for PCR detection of LPs biosynthesis genes in *B. amyloliquefaciens* NCPSJ7.

Gene	Primer	Sequence	Expression Products	Size (bp)	Annealing Tm. (°C)
*srfAA*	SRFAF	5′-TCGGGACAGGAAGACATCAT	Surfactin	201	58 [11]
	SRFAR	5′-CCACTCAAACGGATAATCCTGA			
*sfp*	SFPF	5′-TATATGGACCGCCCGCTTTCTGC	Sfp ^a^	462	60
	SFPR	5′-CCCTTTTCCGGCCTGCTTGATAA			
*bmyB*	BMYBF	5′-GAATCCCGTTGTTCTCCAAA	Bacillomycin	370	55 [11]
	BMYBR	5′-GCGGGTATTGAATGCTTGTT			
*fenD*	FENDF	5′-GGCCCGTTCTCTAAATCCAT	Fengycin	269	58 [11]
	FENDR	5′-GTCATGCTGACGAGAGCAAA			
*ituD*	ITUDF	5′-TTGAAYGTCAGYGCSCCTTT	Iturin	482	58 [24]
	ITUDR	5′-TGCGMAAATAATGGSGTCGT			
*ituC*	ITUCF	5′-CCCCCTCGGTCAAGTGAATA	Iturin	594	58 [24]
	ITUCR	5′-TTGGTTAAGCCCTGATGCTC			

^a^ Sfp, phosphopantheinyl transferase, which is essential for various LPs synthesis [25].

**Table 2:** Antifungal LPs identified after the LC/MS analysis of LP crude.

LPs	Mass Peak	
	[M + H]^+^	[M + Na]^+^
C-13 surfactin	1008	1030
C-15 surfactin	1036	1058
C-12 iturin A	1015	1037
C-13 iturin A	1029	1051
C-14 iturin A	1043	1065
C-15 iturin A	1057	1079
C-16 iturin A	1071	1093
C-16 fengycin A	1463	1485
C-17 fengycin A	1477	1499
C-18 fengycin A	1491	ND

ND, not detected.

## Data Availability

All data generated or analyzed during this study are available upon request from the author.

## Supplementary Materials

The following supporting information can be downloaded at https://www.mdpi.com/article/10.3390/foods11192996/s1: Figure S1—MALDI-TOF-MS analysis of fengycin; Figure S2—LC/MS analysis of LPs crude; Figure S3—HPLC of the iturin A standard and the methanol elution; Figure S4—Mass spectroscopy of the antifungal compound FP1.
