# Supplementary material for: Identification of Lipopeptide Iturin A Produced by Bacillus amyloliquefaciens NCPSJ7 and Its Antifungal Activities against Fusarium oxysporum f. sp. niveum
Source: Foods. 2022 Sep 26;11(19):2996. doi: 10.3390/foods11192996 (PMC9563565; doi:10.3390/foods11192996)
Supplement: Supplementary file 1 [file foods-11-02996-s001.zip › foods-1853707-supplementary.pdf]

Supplementary materials

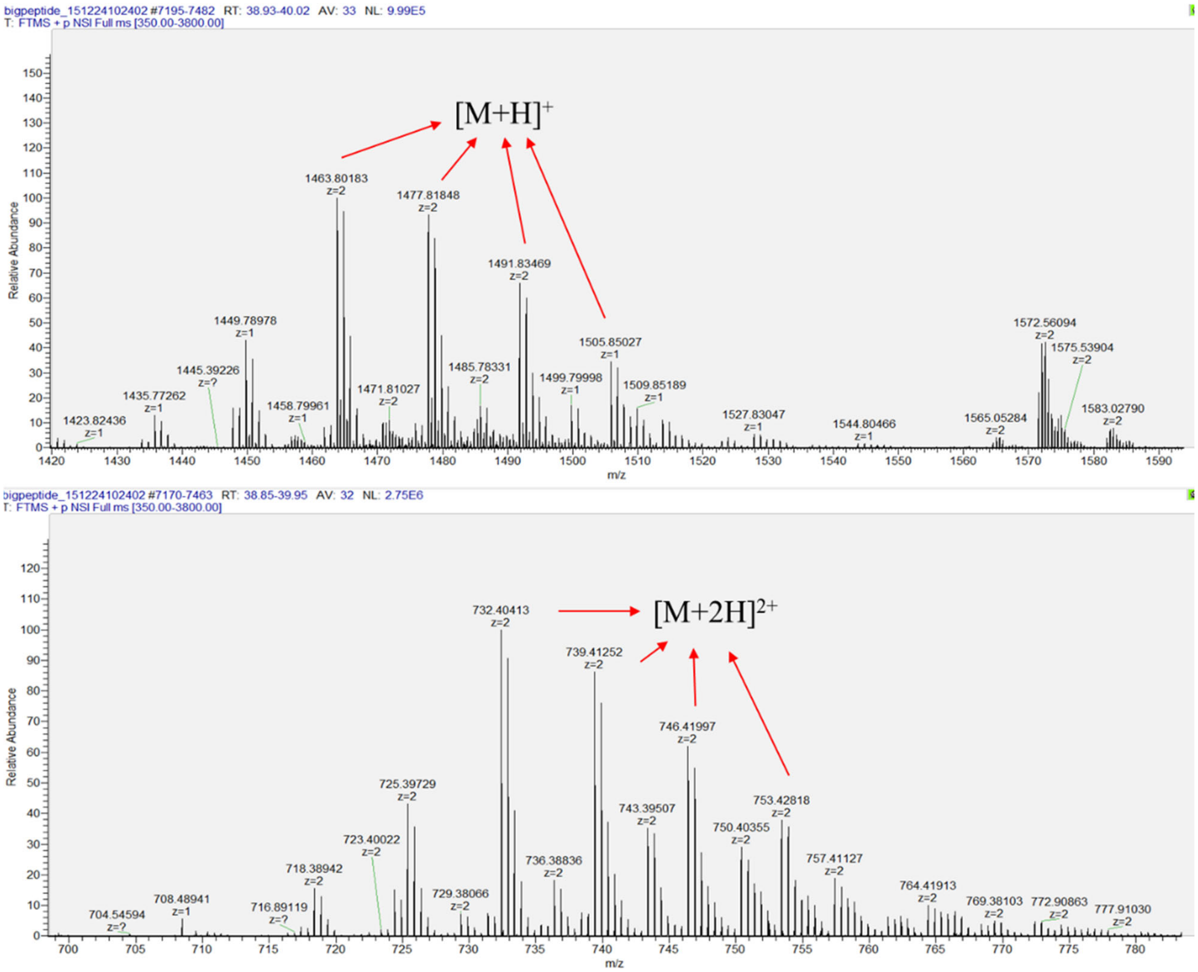

Figure S1. MALDI-TOF -MS analysis of Fengycins.

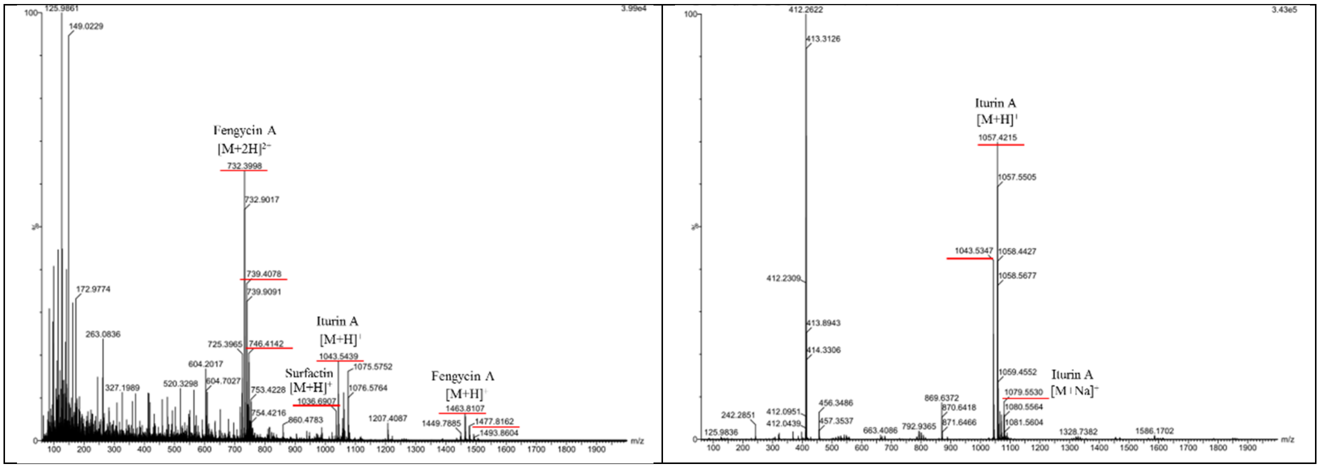

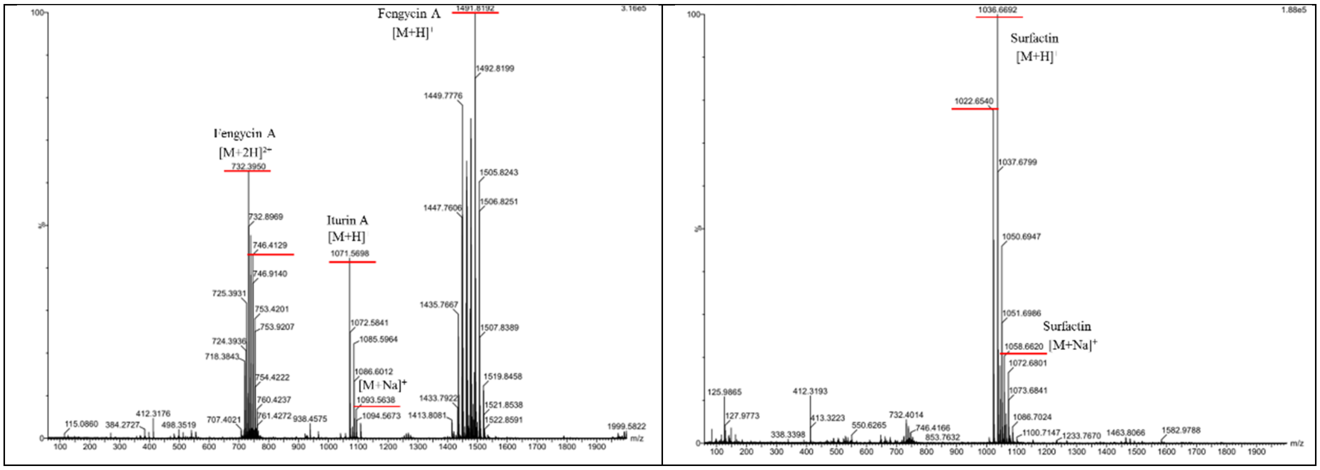

Figure S2. LC/MS analysis of the LPs crude.

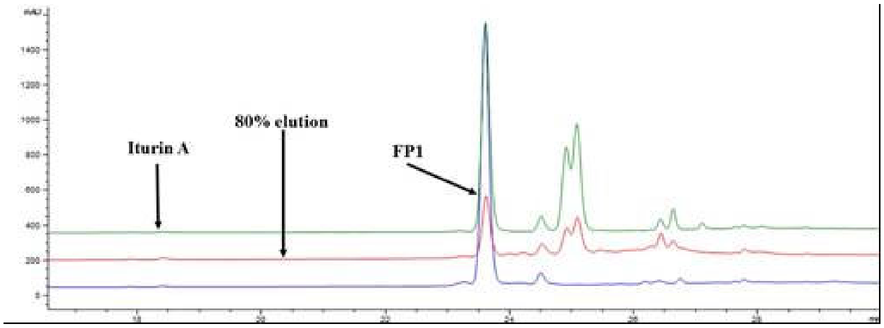

Figure S3. HPLC of the iturin A and the methanol elution.

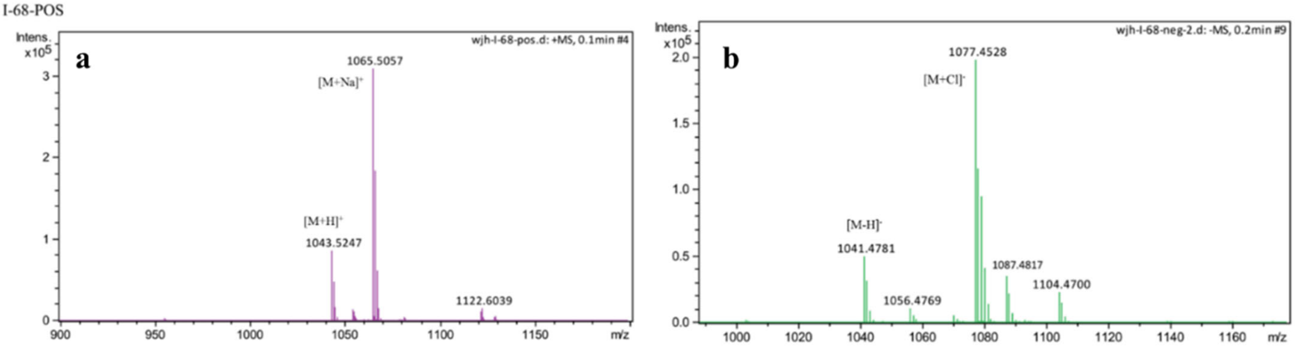

Figure S4. Mass spectroscopy of the antifungal compound FP1. (**a**) positive-ion mode, (**b**) negative-ion mode.
